# Identification and expression analysis of histone modification gene (*HM*) family during somatic embryogenesis of oil palm

**DOI:** 10.1186/s12864-021-08245-2

**Published:** 2022-01-05

**Authors:** Lixia Zhou, Rajesh Yarra, Longfei Jin, Yaodong Yang, Hongxing Cao, Zhihao Zhao

**Affiliations:** Coconut Research Institute, Chinese Academy of Tropical Agricultural Sciences/ Hainan Key Laboratory of Tropical Oil Crops Biology, Wenchang, Hainan 571339 P. R. China

**Keywords:** *HMs*, Somatic embryogenesis, Oil palm, Real-time PCR

## Abstract

**Background:**

Oil palm (*Elaeis guineensis*, Jacq.) is an important vegetable oil-yielding plant. Somatic embryogenesis is a promising method to produce large-scale elite clones to meet the demand for palm oil. The epigenetic mechanisms such as histone modifications have emerged as critical factors during somatic embryogenesis. These histone modifications are associated with the regulation of various genes controlling somatic embryogenesis. To date, none of the information is available on the histone modification gene (*HM*) family in oil palm.

**Results:**

We reported the identification of 109 *HM* gene family members including 48 *HMT*s, 27 *HDM*s, 13 *HAT*s, and 21 *HDAC*s in the oil palm genome. Gene structural and motif analysis of *EgHM*s showed varied exon–intron organization and with conserved motifs among them. The identified 109 *EgHM*s were distributed unevenly across 16 chromosomes and displayed tandem duplication in oil palm genome. Furthermore, relative expression analysis showed the differential expressional pattern of 99 candidate *EgHM* genes at different stages (non-embryogenic, embryogenic, somatic embryo) of somatic embryogenesis process in oil palm, suggesting the *EgHM*s play vital roles in somatic embryogenesis. Our study laid a foundation to understand the regulatory roles of several *EgHM* genes during somatic embryogenesis.

**Conclusions:**

A total of 109 histone modification gene family members were identified in the oil palm genome via genome-wide analysis. The present study provides insightful information regarding *HM* gene’s structure, their distribution, duplication in oil palm genome, and also their evolutionary relationship with other *HM* gene family members in *Arabidopsis* and rice. Finally, our study provided an essential role of oil palm *HM* genes during somatic embryogenesis process.

**Supplementary Information:**

The online version contains supplementary material available at 10.1186/s12864-021-08245-2.

## Background

Histone modifications gene family (*HM*s) play a vital role in plant growth and developmental processes by histone modification processes (methylation, demethylation, acetylation, and deacetylation) either by activating or silencing the gene expression. These four different histone modification processes are regulated by four different *HM* gene family members such as *HMTs* (histone methyltransferases), *HDMs* (histone demethylases), *HATs* (histone acetylases), and *HDACs* (histone deacetylases) [1, 2]. Each of the *HM* gene family members also consisted of a varied number of subfamilies with different domain structures. The *HMTs* contained *SDG* (set domain group) and *PRMT* (protein arginine methyltransferases) sub-families. The *HDMs* contained *HDMA* (SWIRM and C-terminal domain) and *JMJ* (JmjC domain-containing proteins) sub-families. The *HATs* contained *HAG* (GCN5-, ELP3-, and *HAT*1-like histone acetylases domain structure), *HAM* (MOZ-YBF2 domain), *HAC* (p300/CREB-binding protein structure), and *HAF* (TATA-binding protein-associated factors TAF_II_250) sub-families. The *HDAC*s contained *HDA* (RPD3/HDA1 superfamily), *SRT* (silent information regulator 2), and *HDT* (HD2 families) sub-families [3–7]. Numerous previous studies described the role of *HM* gene family members in regulating the vegetative and reproductive growth, biotic and abiotic stress responses, stress-related hormone signaling [4, 8–16]. Various genome-wide studies revealed the occurrence of a varied number of *HM* gene family members in various plants including a total of 198 *HM*s in *Malus domestica * [8]; 125 *HM*s in *Lycopersicum esculentum * [17]*;* 136 *HM*s in *Citrus sinensis * [18]; 87 *HM*s in *Litchi chinensis* [9]. However, to date, none of the studies were published on genome-wide studies of *HM* gene family members in oil palm genome.

African oil palm (*Elaeis guineensis*, Jacq.) is the most promising and productive oil crop to accomplish the increasing demand for vegetable oils around the world [19, 20]. The vegetative propagation for large-scale production of oil palm plants is not possible due to the absence of auxiliary shoots and traditional seed propagation is hampered by the low seed germination rate [21, 22]. Moreover, genetic improvement of oil palm plants via seed propagation is the most complicated [22]. A promising substitute for the large-scale production of oil palm seedlings is the extensive micropropagation of plants via tissue culture method of approach i.e. somatic embryogenesis (SE) [22, 23]. In plants, somatic embryogenesis is the method of producing somatic embryos under in vitro conditions without the fusion of the gametes [24]. Somatic embryogenesis is the reliable and powerful biotechnological approach for the micropropagation of plants with low seed germination rates as well as long reproductive cycles [25]. However, the somatic embryogenesis response is differed from species to species depending on their totipotency capacity. Though somatic embryogenesis protocols were well established for the propagation of oil palm, it’s essential to understand metabolic, genetic, epigenetic, morphogenetic factors that boost the somatic embryogenesis process in oil palm plants. Moreover, epigenetic mechanisms such as methylation, demethylation, acetylation, and deacetylation are regulating the gene expression that modulates the capacity of somatic embryogenesis during tissue culture [26]. The epigenetic mechanisms that regulate the gene expression at somatic embryogenesis have not been addressed much in oil palm. Identification of genes that are responsible for regulating the epigenetic mechanisms during somatic embryogenesis of oil palm is vital for a better understanding of SE process. Up to now, none of the studies were reported on histone modification (*HM*) gene family members in oil palm during different somatic embryogenesis phases.

In this study, we identified a total of 109 *HM* gene family members (48 *HMT*s; 27 *HDM*s; 13 *HAT*s, and 21 *HDAC*s) in the oil palm genome via the bioinformatics approach. We also analyzed the *EgHM*s gene structure, motif analysis, phylogenetic analysis, synteny, promoter analysis, subcellular localization, and their location on 16 chromosomes in oil palm genome. Further, we analyzed the expression patterns of all identified *EgHM*s at different stages of the somatic embryogenesis process of oil palm.

## Results

### Identification of *HM* gene family in oil palm genome

In this investigation, a total of 109 *EgHM* gene family members such as 48 of *HMT*s (histone methyltransferases); 27 of *HDM*s (histone demethylases); 13 of *HAT*s (histone acetylases), and 21 of *HDAC*s (histone deacetylases) were successfully identified in the oil palm genome via genome-wide analysis. All the *EgHM* family members are categorized into 11 subfamilies (*SDG*, *PRMT*, *HDMA*, *JMJ*, *HAG*, *HAM*, *HAC*, *HAF*, *HAD*, *SRT* & *HDT*) based on their protein domain architecture. The *HMT* family is included with 39*SDG*s and 9*PRMT*s; *HDM* family is contained with 3*HDMA*s and 24*JMJ*s; *HAT* family is included with 4*HAG*s, 2*HAM*s, 6*HAC*s, and 1*HAF*; *HDAC* family included with 15*HAD*s, 3*SRT*s, and 3 *HDT* sub-families. All of the gene IDs of *EgHM* family members were provided in Supplementary Table 1. The pI and Molecular weight of the oil palm *HM* family members were ranged from 4.57 to 9.95 and 12.3 to 273.3 respectively (Supplementary Table 1). The *EgHMs* are predominantly localized to the cytoplasmic regions (Supplementary Table 2).

### Gene structure and conserved motif analysis of *EgHM* family members

**Fig. 1:**
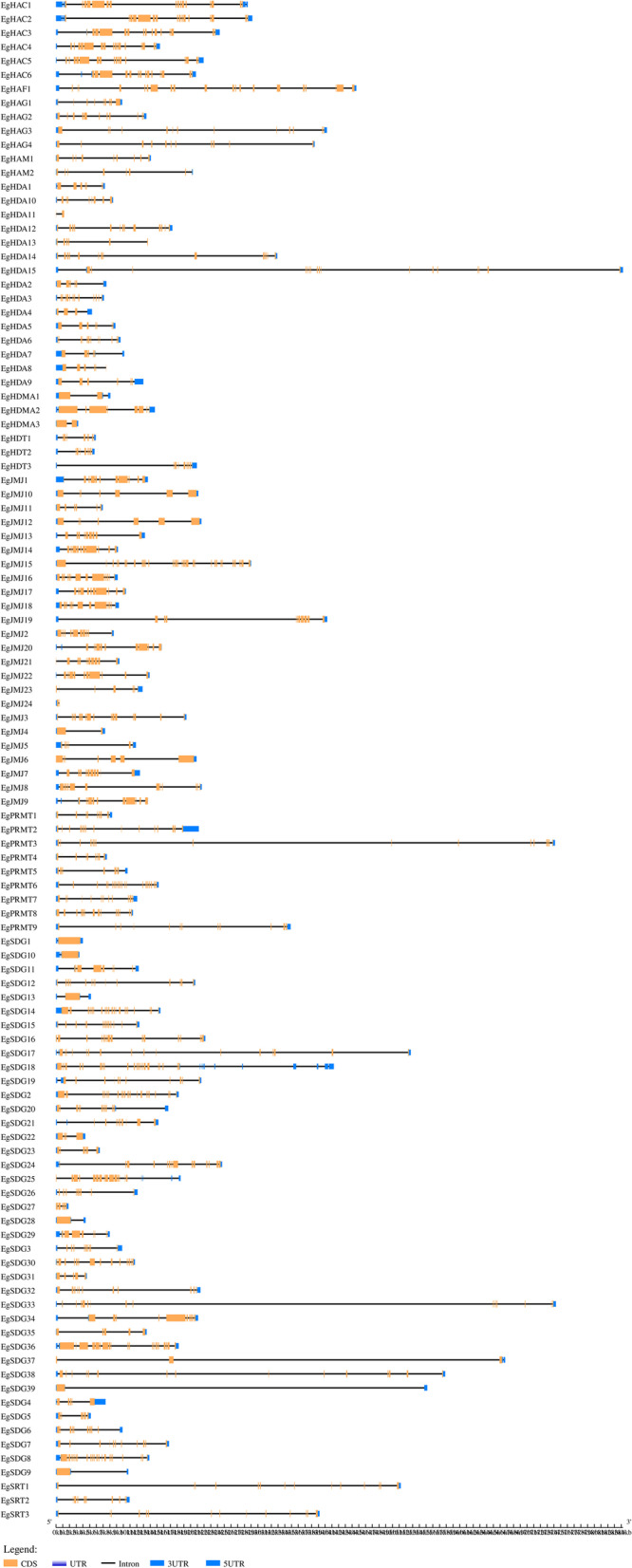
Gene structural organization of 109 *EgHMs*. Coding sequences (CDS) are represented by orange color blocks; 3’ & 5’ UTRs are re*g*ions are represented by blue color blocks; intron r*eg*ions are represented by black color blocks in the diagram

**Fig. 2:**
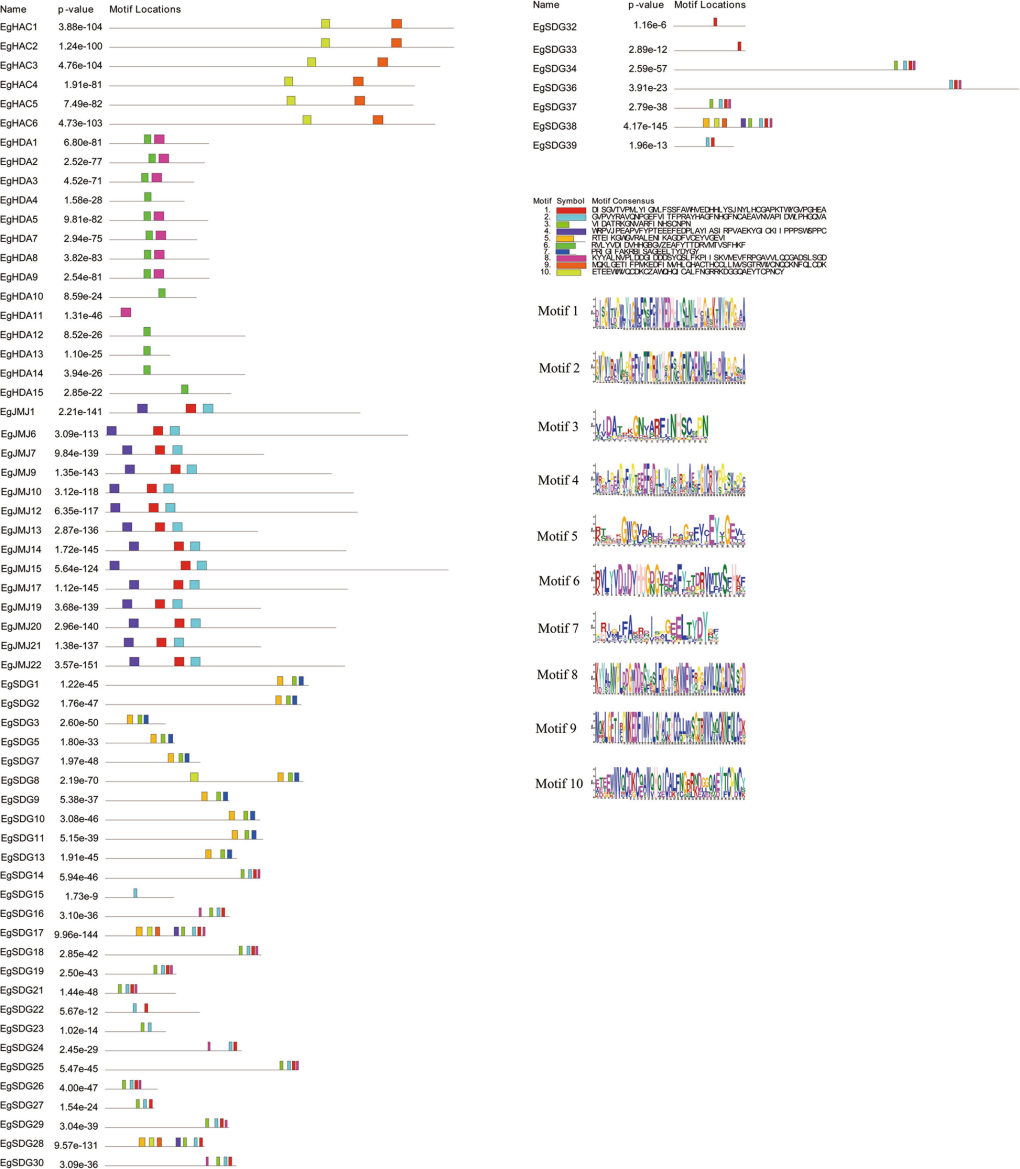
Occurrence of conserved motifs in *EgHM* proteins. The MEME tool was used to identify the ten numbers of motifs in *EgHM* proteins. Each motif in *EgHM* proteins has shown with different colors. The sequence logo of each motif represents the abundance of each amino acid in their motifs

We analyzed the gene structure of all *EgHM* gene family members using the Gene Structure Display Server tool 2.0 (http://gsds.cbi.pku.edu.cn/). We found the occurrence of varied numbers (2–34) of exons among the *HM* gene family m*embers* (Fig. 1)*.* The highest number (34) of exons was observed in *EgHMT* members i.e. *EgSDG*18 and the least number (2) of exons were observed in *EgSDG39* (Fig. 1). We found10 conserved motifs among the 109 *HM* gene family of oil palm (Fig. 2).

### Chromosomal distribution of *EgHM* members in oil palm genome

**Fig. 3:**
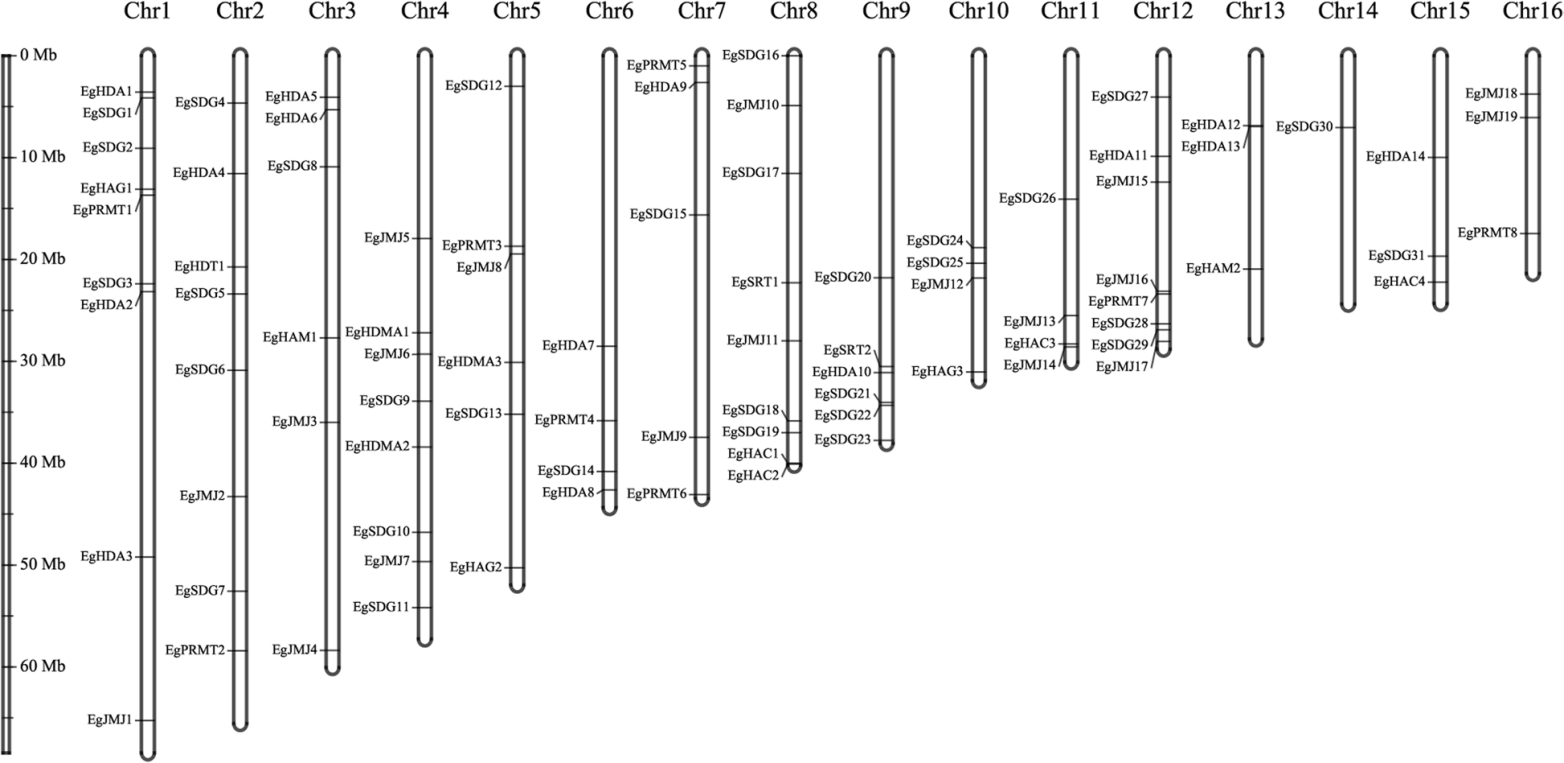
Chromosomal distribution of *EgHM* genes across 16 chromosomes of oil palm genome. Chromosome numbers are indicated on the top of each chromosome. The vertical greyscale on the left side represents the length of the oil palm chromosomes

The chromosomal distribution of 109 *EgHM* gene family members was also examined across the 16 chromosomes of the African oil palm genome. The *EgHM* gene family members were unevenly distributed on the chromosomes (Fig. 3). Among the identified 109*EgHMs,* only 86 were mapped across the 16 chromosomes (Fig. 3). We have not observed the mapping of 26 *EgHM* members on any of the chromosomes. Chromosomes 1 and 9 had the highest number (9) of *EgHM* family genes, whereas chromosome 14 had only one *EgHM* family gene (Fig. 3). Apart from chromosome 14, all of the remaining chromosomes at least contained 3 or more *EgHM* family genes.

### *EgHM* gene duplication in oil palm genome

**Fig. 4:**
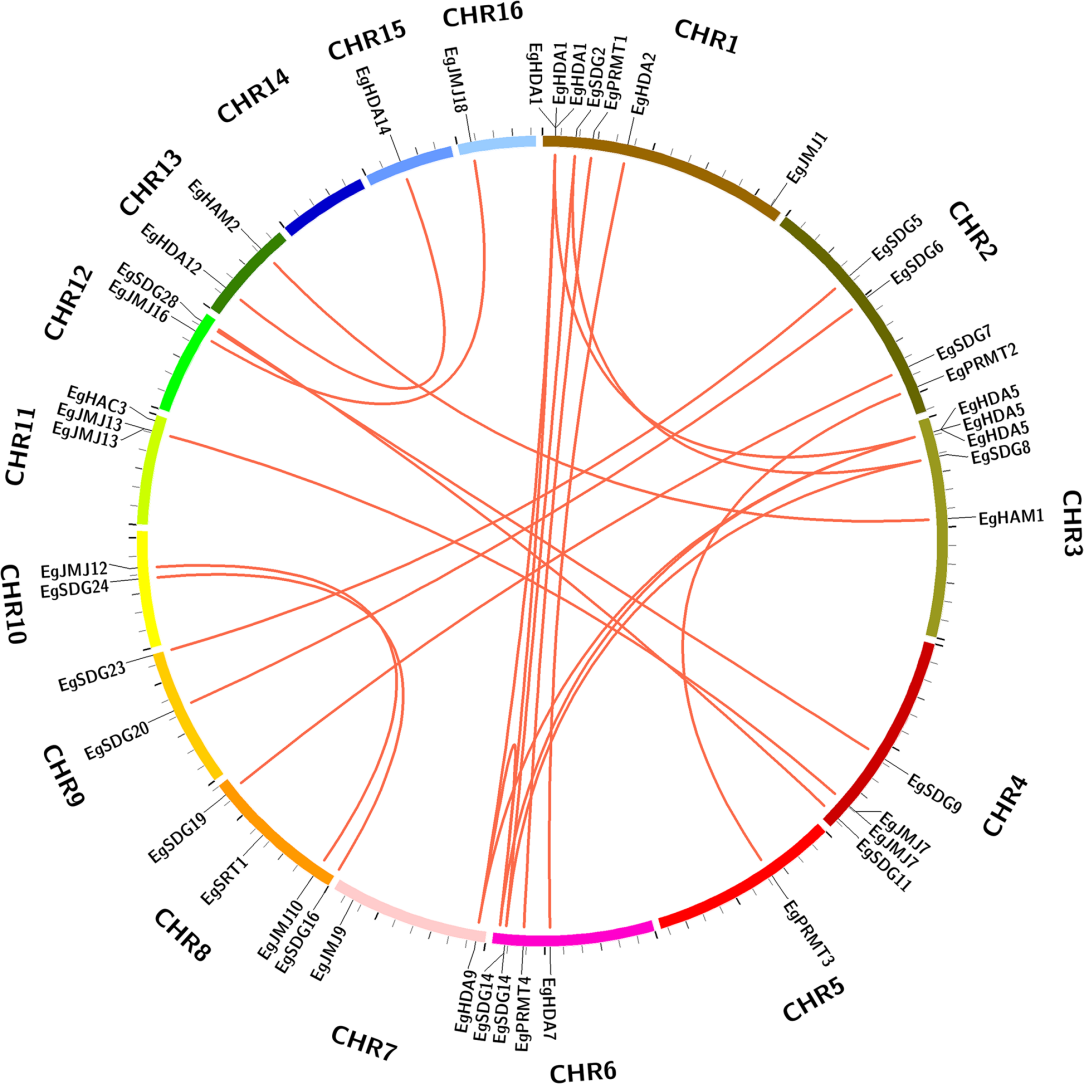
Gene duplication events and interchromosomal relationships between *EgHM* genes in oil palm genome. A total of 44 duplicated *EgHM* gene pairs were found across 16 chromosomes *vi*a MC ScanX tool and linked by the red lines inside the circle view. Each chromosomal block was represented with a different color

To know the expansion of the *HM* gene family in oil palm genome, we generated a gene duplication event diagram for duplicated blocks using a Circos algorithm. In a total, 37 pairs of Eg*HMs* were identified from 16 chromosomes of oil palm, including 14 pairs of *EgSDG*s; 4 pairs of *EgPRMT*s; 6 pairs of *EgJMJ*s; 2 pairs of *EgHAM*s; 1 pair of *EgHAC*s; 9 pairs of *EgHDA*s, and 1 pair of *EgSRT*s (Fig. 4). The paired *EgHM* duplicated genes were all located in different chromosome blocks of oil palm genome (Fig. 4). Moreover, Chromosome 1 and Chromosome 6 had 6 and 5 number of duplicated genes respectively. However, chromosome 14 block had no duplicated *HM* genes (Fig. 4). These results demonstrated the expansion of *EgHM* gene family that occurred through these duplicated regions.

### Phylogenetic analysis between oil palm, rice, and *Arabidopsis HM* gene family

**Fig. 5:**
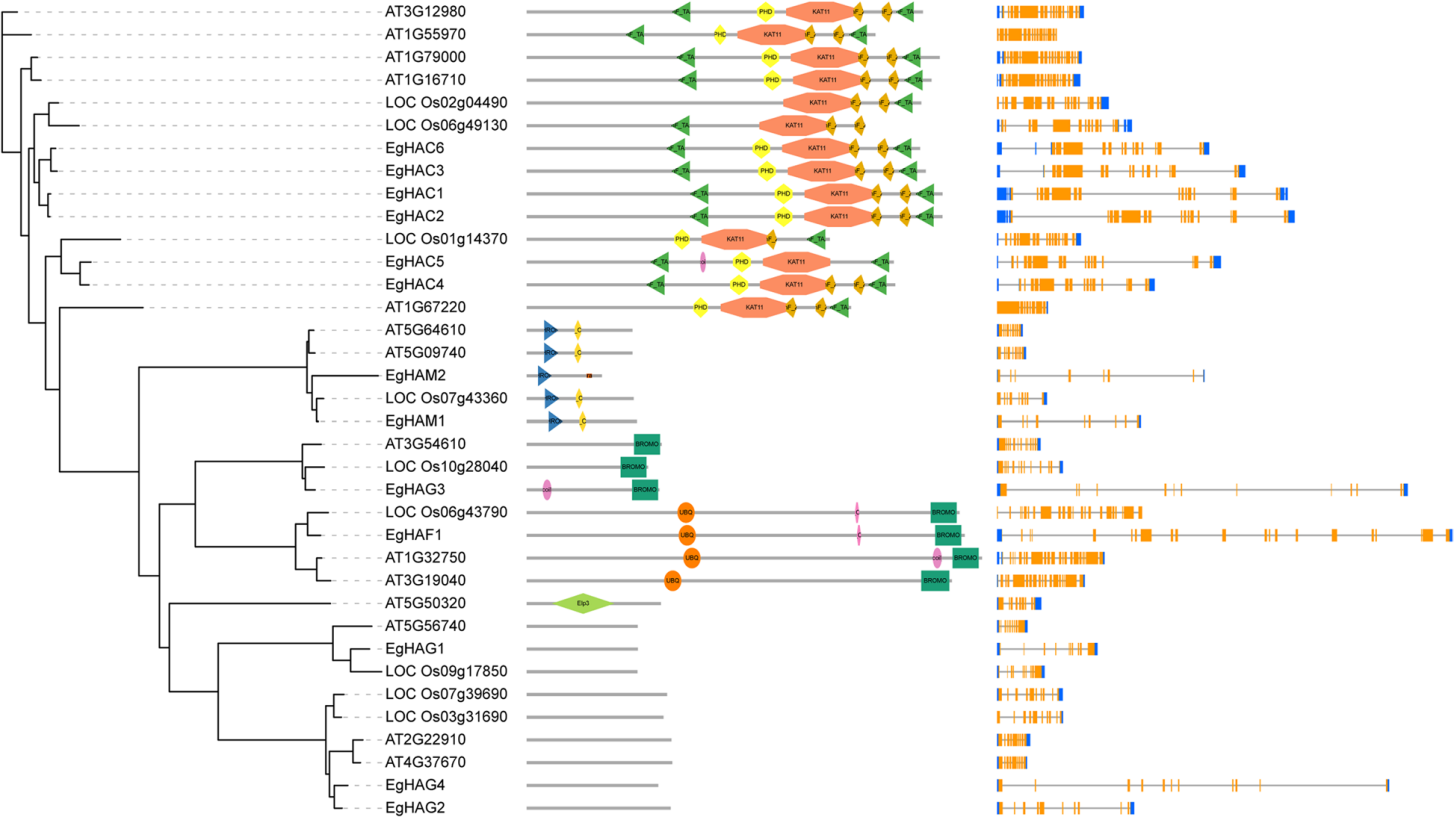
Phylogenetic analysis of *EgHAT* family with rice and *Arabidopsis HAT* family genes. Domain composition and gene structure of oil palm, rice, and *Arabidopsis HAT* members were also shown in the middle and right sides respectively. Domains were represented by different colours. Exon(s) and intron(s) were shown in orange boxes and black lines respectively. The UTR regions (5^/^ & 3^/^) were represented by blue boxes

**Fig. 6:**
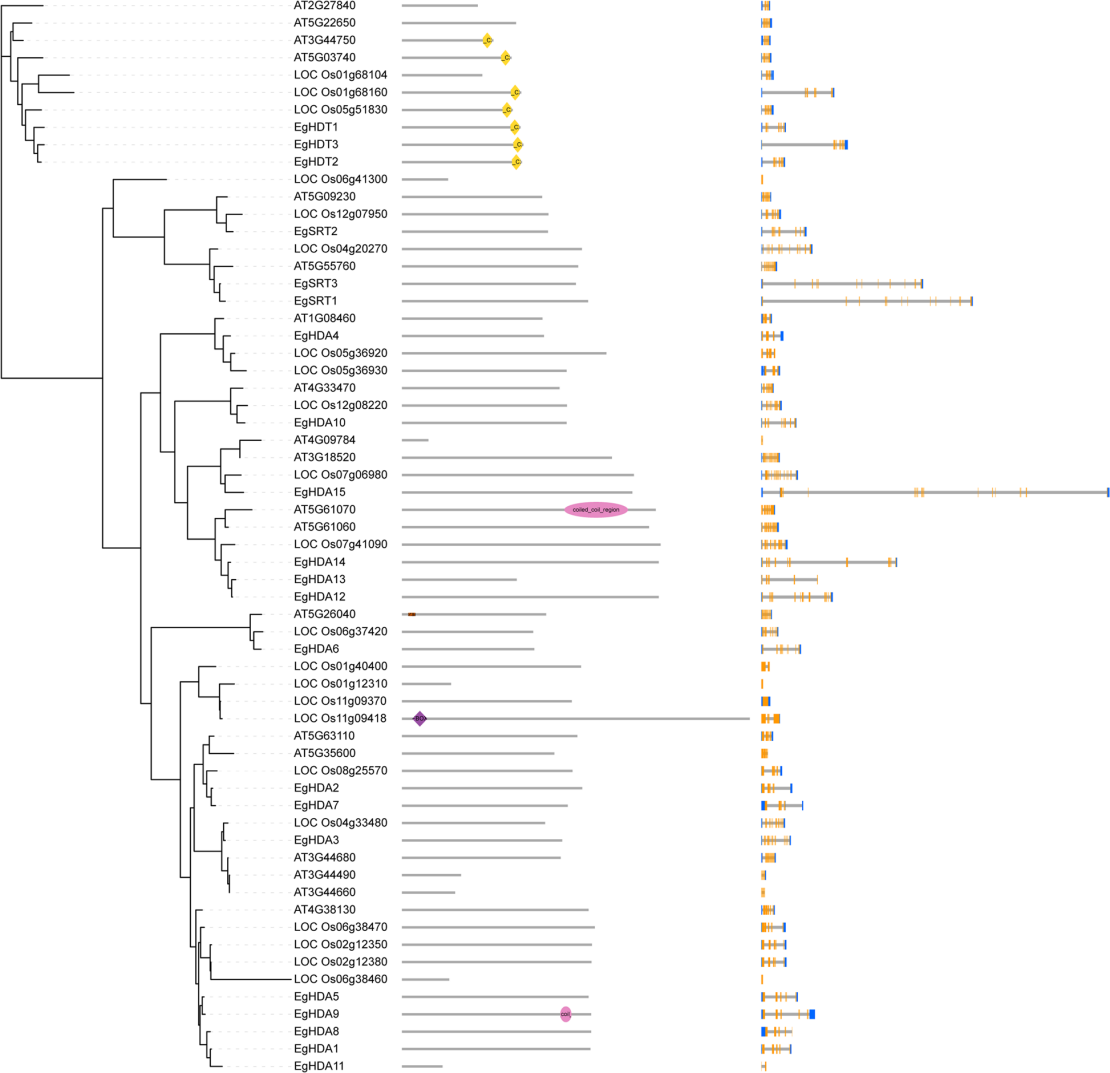
Phylogenetic analysis of *EgHDACs* with rice and *Arabidopsis HDAC* genes. Domain composition and gene structure of oil palm, rice, and *Arabidopsis HDAC* members were also shown in the middle and right sides respectively. Domains were represented by different colours. Exon(s) and intron(s) were shown in orange boxes and black lines respectively. The UTR regions (5^/^ & 3^/^) were represented by blue boxes

**Fig. 7:**
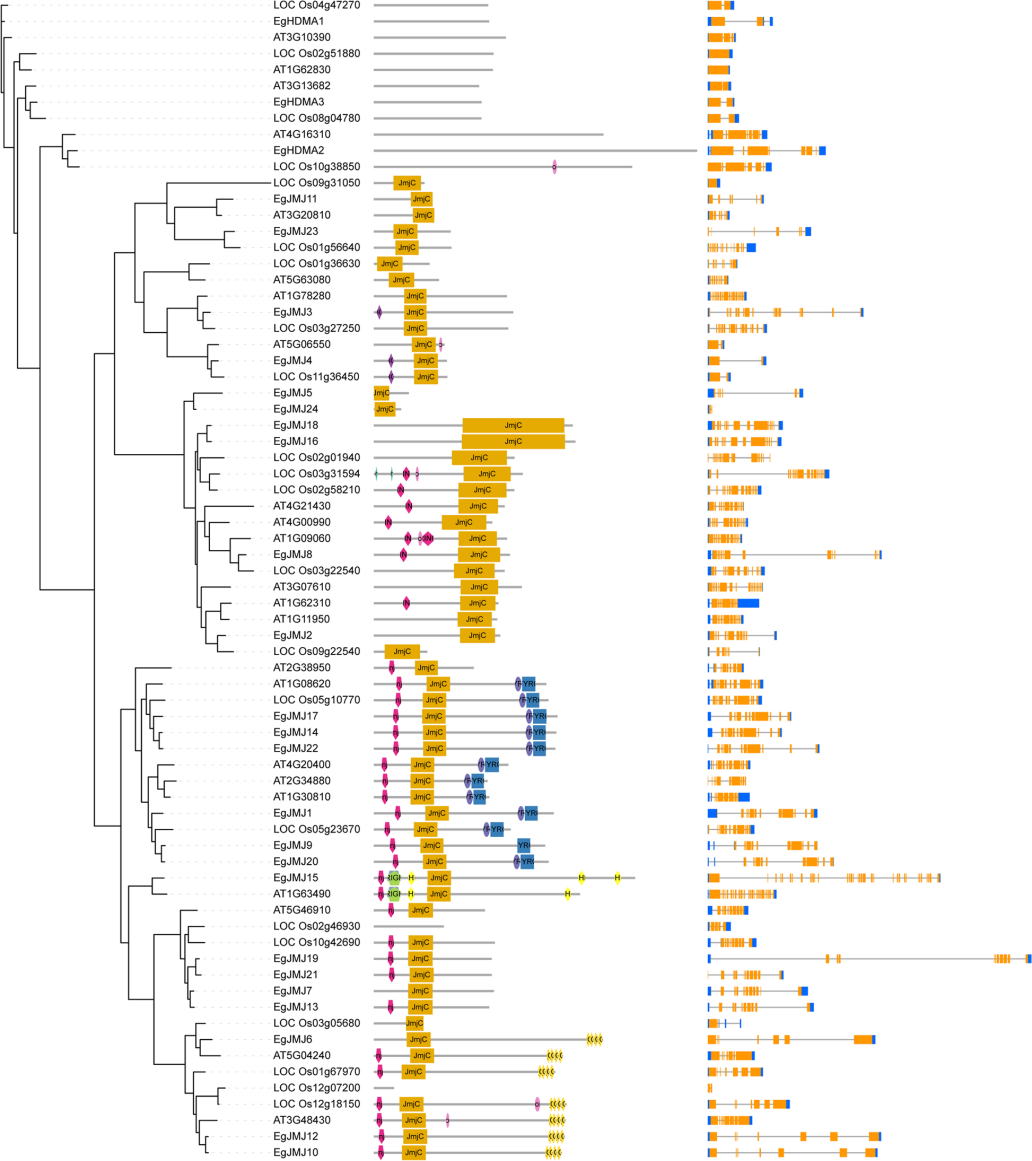
Phylogenetic analysis of *EgHDMs* with rice and *Arabidposis HDM* genes. Domain composition and gene structure of oil palm, rice, and *Arabidopsis HDM* members were also shown in the middle and right sides respectively. Domains were represented by different colours. Exon(s) and intron(s) were shown in orange boxes and black lines respectively. The UTR regions (5^/^ & 3^/^) were represented by blue boxes

**Fig. 8:**
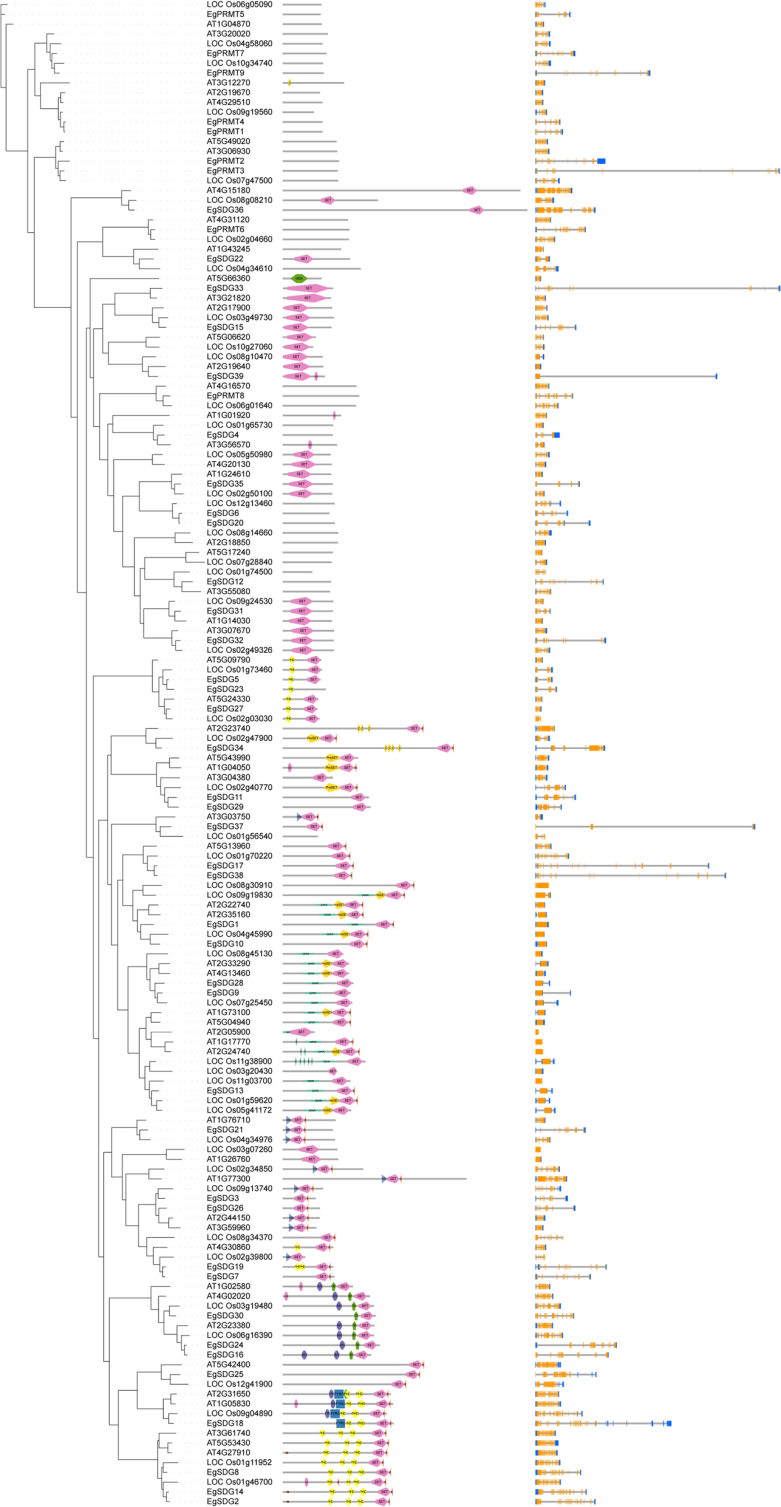
Phylogenetic analysis of *EgHMTs* with rice and *Arabidopsis HMT* genes. Domain composition and gene structure of oil palm, rice and *Arabidopsis HMT* members were also shown in the middle and right sides respectively. Domains were represented by different colours. Exon(s) and intron(s) were shown in orange boxes and black lines respectively. The UTR regions (5^/^ & 3^/^) were represented by blue boxes

To elucidate the evolutionary relationship between oil palm, rice, Arabidopsis, we generated the rooted phylogenetic trees for each *HM* gene family (*HAT, HDAC, HDM,* and *HMT*) (Figs. 5, 6, 7 and 8). The phylogenetic tree for subfamilies of each HM gene family was classified and clustered into diverse trends. The phylogenetic tree of HAT family showed that all the *HAG*, *HAM*, *HAC,* and *HAF*s of oil palm, rice, and *Arabidopsis* were not clustered together and they clustered in a species-specific manner and also in mixed type (Fig. 5). For *HDAC* family, *HDT*s and *SRT*s clustered in a species-specific manner, whereas *HDA*s clustered together (Fig. 6). The phylogenetic tree of *HDM*s (*HDMA*s and *JMJ*s) also showed a different trend with a species-specific type of clustering (Fig. 7). The phylogenetic tree of *HMT* family revealed that all the *SDGs* and *PRMTs* genes were clustered together (Fig. 8). Altogether, our results indicated that there is a clear evolutionary relationship and diversification between oil palm, rice, and *Arabidopsis HM* gene family members.

### Expression levels of ***EgHM***s in different somatic embryogenic stages (Embryogenic calli, Non-embryogenic calli, and somatic embryos)

**Fig. 9:**
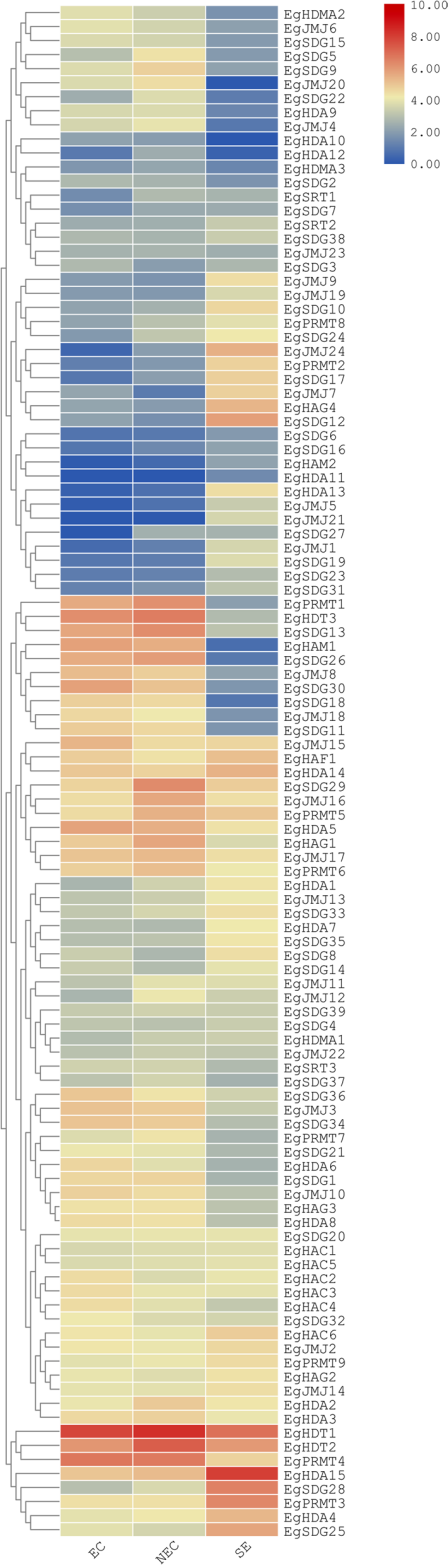
Heat map showing the expression profiles of *EgHM* genes in various stages (EC: Embryogenic calli; NEC:Non-embryogenic calli; SE:Somatic embryos)) of oil palm somatic embryogenesis. The color scale indicates the level of *EgHM* genes expression. The raw transcriptome data was downloaded from NCBI database (Accession number is PRJNA699335)

Based on the available transcriptome data of oil palm EC, NEC, and SE (https://www.ncbi.nlm.nih.gov/bioproject/PRJNA699335) stages were downloaded from the NCBI website and analyzed the expression levels of all identified 109 *EgHM* gene family members. The transcript abundance of *EgHM*s in various stages of oil palm somatic embryogenesis was analyzed by generating the heatmap with the help of FPKM values. As shown in Fig. 9, *EgHM* gene members showed differential expression in different stages of somatic embryogenesis of oil palm. However, most of the genes were down-regulated in all three stages of somatic embryogenesis (Fig. 9). Moreover, six members of *HMTs (EgSDG*13, *EgSDG*26, *EgSDG*30, *EgSDG*34, *EgPRMT*1 and *EgPRMT*4), two members of *HDM*s (*EgJMJ*15 and *EgJMJ*17); on member of *HAT*s (*EgHAM*1); and four members of *HDAC*s(*EgHDA*5, *EgHDT*1, *EgHDT*2, *EgHDT*3) are expressed during both EC and NEC stages (Fig. 9). The *HDAC* genes *EgHDT*1 and *EgHDT*2 were showed expression in all three stages of somatic embryogenesis. A total of seven *HM* members including *EgJMJ*24, *EgHAG*4, *EgSDG*12, *EgSDG*25, *EgSDG*28, *EgPRMT*3 and *EgHDA*4 were only expressed in SE stage. Though *EgHDA*15 expressed in all three stages of somatic embryogenesis, it is highly upregulated in SE stage. None of the *HM* members were highly and specifically expressed either in EC or NEC stage. *EgHDT*1 is highly expressed in EC and NEC stages of somatic embryogenesis (Fig. 9). Our results elucidated the role of specific *EgHM*s during the conversion of NEC to SE during oil palm somatic embryogenesis.

### Real time-PCR expression analysis of candidate *EgHM* family genes

**Fig. 10:**
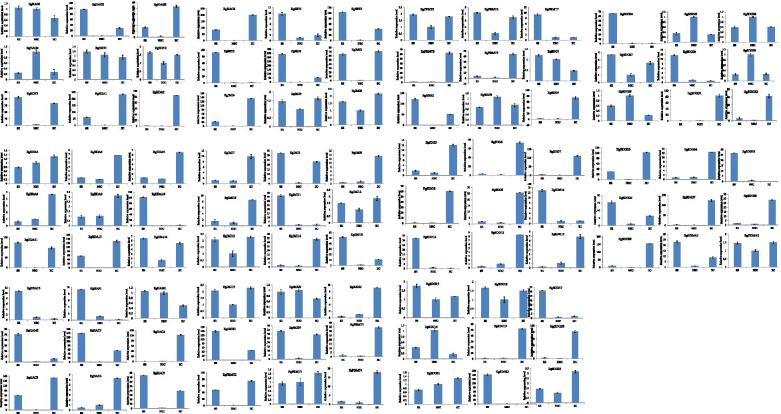
Relative expression analysis of 99 *EgHM* candidate genes in various stages of somatic embryogenesis in oil palm. The X-axis represents different stages (SE, Somatic embryos; NEC, non-embryogenic calli; EC, embryogenic calli); Y-axis represents relative expression of specific *EgHM* gene. Data represent the mean ± SE of three replicates. Asterisks represent significant differences at *P* ≤ *0.05*(*) and *P* ≤ *0.01*(**)

A total of 99 *EgHM* genes were selected and analyzed their relative expression levels during different stages (NEC, EC & SE) of somatic embryogenesis of oil palm through q-RTPCR. Our results revealed the varied expression levels of selected *EgHM*s during various stages of somatic embryogenesis. The relative expression of *HMT*s (*PRMT*s & *SDG*s) was significantly higher in EC and SE stages than NEC stage (Fig. 10). *EgPRMT*2 is highly expressed in EC and SE stages, whereas *EgPRMT*8 has shown the highest expression in SE stages of somatic embryogenesis (Fig. 10). *EgPRMT*2 & 5 have shown similar expression in all three stages of somatic embryogenesis. *EgSDG*24, 28, 35 were highly expressed in SE stage and *EgSDG*18 has shown the highest level of expression in NEC stage. *EgSDG*19, 20, and 37 have shown significantly higher expression in EC stage (Fig. 10). The relative expression of *HDM*s (*HDMA*s & *JMJ*s) was significantly higher in EC and SE stages, whereas *EgJMJ*20 has shown the highest expression in NEC stage and also in SE stage (Fig. 10). The relative expression of *HAT*s was also significantly higher in both EC and SE stages. Some of them have shown the highest expression in specific stages (either in EC or SE) (Fig. 10). The relative expression of a majority of *HDAC*s is also higher in EC and SE stages than NEC. *EgSRT1* and *EgHDA*15 were highly expressed in SE stage (Fig. 10). Taken together, all these results indicate the potential role of some *EgHM*s in somatic embryogenesis of oil palm.

## Discussion

Somatic embryogenesis is an important tissue culture approach for plant regeneration and it incites various epigenetic changes such as histone modifications (methylation, demethylation, acetylation, and deacetylation [27, 28]. During the somatic embryogenesis process, various histone modifications are potentially regulated [27, 29]. The histone modifications activity leads to the altered gene expression of somatic embryogenesis process [27]. These histone modification changes regulated by various genes during somatic embryos potentially affect the development of somatic embryos. It’s necessary to know the information of various genes that regulate the histone modifications during somatic embryogenesis. To date, none of the studies provided brief information on histone modification genes of oil palm during somatic embryogenesis process. To the best of our knowledge, this is the first report on the identification and characterization of *HM* genes in oil palm during somatic embryogenesis process. In our study, we identified a total of 109 HM genes including 48 of *HMT*s (histone methyltransferases); 27 of *HDM*s (histone demethylases); 13 of *HAT*s (histone acetylases), and 21 of *HDAC*s (histone deacetylases) through genome-wide analysis and analyzed their expression patterns during different stages (NEC, EC, and SE) of somatic embryogenesis process of oil palm. We reported a comprehensive study including gene structural organization, motif composition, and location on chromosomes, duplication events, and phylogenetic analysis of all of identified 109 *EgHM*s.

The number (109) of identified *HM*s in oil palm genome were neither more nor lesser than the identified *HM* gene family members in other crops including *Malus domestica* (198*HM*s) [8]; *Lycopersicum esculentum* (125 *HM*s) [17]; in *Citrus sinensis* (136 *HM*s) [18]; *Litchi chinensis* (87 *HM*s) [9], indicating that the variance in occurrence of several *HM* genes is not related with the genome size of species. The identified *EgHM* gene family members were divided into four categories and then 11 sub-families, which is similar to the classification of *HM* genes published previously in other crops [8, 9, 17, 18]. The diversification of the gene family is majorly associated with the intron–exon organization (gain or loss) of genes [30]. Our study also demonstrated that the *EgHM* genes with similar structural organization were clustered together, whereas others with different structural organizations were grouped distantly. Our findings have coincided with the previous studies genome-wide identification studies of *HM* gene families in the apple and citrus genome [8, 18]. The typical domains in gene clusters of *HM* gene families are conserved in most of the crops [8, 17, 18]. Moreover, oil palm *HM* family genes also contained the same typical domain structure as similar to other plant *HM* gene families [8, 17, 18].

The phylogenetic analysis of oil palm *HM* gene family with *Arabidopsis* and rice was analyzed for each family (*HMT*, *HDM*, *HAT,* and *HDAC*). They were clustered in a species-specific manner which was slightly different from the previous reports on HM gene families in various crops [8, 17]. Our results support the diversification of oil palm *HM* gene family from other crops, however, future phylogenetic studies are needed to know the evolutionary relationship with other closely related palm plants. The chromosomal distribution of all 109 *HM* genes was uneven across 16 chromosomes of oil palm and our results are coinciding with the previous reports on *HM* gene family distribution on apple and citrus genome [8, 17]. Gene duplication plays an essential role in making the complexity of genomes and also in evolutionary lineages [31–33]. Our results also demonstrated the tandem duplication of *EgHM*s in oil palm genome during the evolutionary process. These findings are consistent with the expansion of *HM* gene families during the evolution of the apple genome [8].

The epigenetic events trigger the expression of a specific set of genes that are responsible for cellular totipotency [34]. The histone modifications modulate the expression of genes responsible for somatic embryogenesis [29, 34]. In our study, several genes encoding histone methylation, demethylation, histone acetylation, and histone deacetylation were identified through genome-wide study and analyzed their expression analysis during different stages (NEC, EC & SE) of somatic embryogenesis. In this study, several *EgHMs* displayed their differential expression between different stages of somatic embryogenesis of oil palm as shown in Fig. 10. Based on qPCR data, *EgPRMT*2, *EgPRMT*3, *EgPRMT*5, *EgPRMT*6, *EgSDG*1, *EgSDG*3, *EgSDG*15, *EgSDG*16, *EgSDG*21, *EgSDG*23, *EgSDG*26, *EgSDG*27, *EgSDG*33, *EgHDMA*2, *EgJMJ*2, *EgJMJ*5, *EgJMJ6*, *EgJMJ*8, *EgJMJ1*2, *EgJMJ*13, *EgJMJ*19, *EgJMJ*24, *EgHAM*1, *EgHAC*3, *EgHAC*5, *EgHAC*6, *EgHAG1, EgHAG*2, *EgHAG*3, *EgHDT*1, *EgHDT*2, *EgHDT*3, *EgHDA*11 & *EgHDA*14 showed their expression levels during both embryogenic calli and somatic embryo stages, revealed their prominent role in somatic embryogenesis. These identified *EgHM*s may provide insightful information to elucidate the molecular mechanisms associated with the somatic embryogenesis of oil palm.

## Material and methods

### *EgHM*s identification in oil palm genome

The pfam database was used to identify the *EgHM* gene family members using the Hidden Markov Model profiles of published IDs of each type (*HMT*, *HDM*, *HAT* & *HDAC*) of *HM* genes [17, 18]. The pfam IDs belongs each type of *HM* genes were used as a query to search for HM gene members in oil palm genome database [35] using HMMER3.0 tool. Further, we also retrieved the unavailable sequences of *EgHMs* using the known *HM* gene sequences of *Arabidopsis* (http://www.arabidopsis.org/) and *Oryza sativa* (http://rice.plantbiology.msu.edu/) through blast search in oil palm genome database (http://palmxplore.mpob.gov.my/palmXplore/).

We also predicted the coding sequence length of each *HM* gene family member using Blastn search against oil palm genome database. The identified putative oil palm *HMs* genes, such as *HMTs* (*SDGs* and *PRMTs*), *HDMs* (*HDMAs* and *JMJs*), *HATs* (*HAGs*, *HAMs*, *HACs*, and *HAFs*), and *HDACs* (*HDAs*, *SRTs*, and *HDTs*) were identified finally based on the highly conserved domains. Additionally, the M.wt, pI values of oil palm *HM* gene members were also determined with the help of ExPASy (https:// web. expasy. org/ compu te_ pi/). The online tool “CELLO” (http://cello.life.nctu.edu.tw/) was further used to predict the subcellular localization of all oil palm *HM* gene family members.

### Gene structure, Conserved motifs analysis of *EgHM*s

The 109 *EgHM* genes structural analysis (intron–exon organization) was analyzed by the Gene Structure Display Server (http://gsds.cbi.pku.edu.cn/). The conserved motifs analysis of all identified *EgHM* proteins were investigated by MEME tool (http:// meme- suite. org/ tools/ meme)**.**

### *EgHM* gene duplications, phylogenetic relationships, and their distribution on chromosomes

We explored the duplications of 109 *EgHM* gene family members in oil palm genome using the MCScanX tool with default parameters [36, 37]. Further, we mapped the location of all 109 *EgHM*s across 16 chromosomes of oil palm from the available genome database of oil palm. We also generated the phylogenetic tree for each type (*HMT*, *HDM*, *HAT* & *HDAC*) of oil palm *HM*s with *HM* genes *Arabidopsis* and *Oryza sativa* using MEGA 7.0 [38] by Maximum Likelihood method, with a bootstrap value of 1000 replications.

### Plant materials

The Oil palm (*Elaeis guineensis;* pisifera, thin-shelled African oil palm) plants were grown in the coconut field of Coconut Research Institute, Chinese Academy of Tropical Agricultural Sciences, Wenchang, China. All the plants were grown under institutional regulatory issues. All the plant materials are collected by the corresponding author of this research work. The immature zygotic embryos were selected as explants for oil palm tissue culture and followed the method as described by Silva et al. (2012) [39] with few modifications. The calli (non-embryogenic or embryogenic) was induced from immature zygotic embryos on callus induction media (CIM).The explants were cultured on CIM: (1/2 MS medium supplemented with 30 mg/l picloram, 100 mg/l casein hydrolysate, 500 mg/l L-glutamine, 200 mg/l aspargine, 200 mg/l arginine, 2 mg/l glycine, 100 mg/l adenine sulfate, 100 mg/l citric acid, 100 mg/l ascorbic acid, 30,000 mg/l sucrose, and 3,000 mg/l Phytagel). The EC and NEC were induced after 3 months of repeated subcultures. The Somatic embryogenesis (SE) was induced by transferring EC to somatic embryo induction medium (SIM: CIM without picloram). The somatic embryo was induced in SIM after four months of culture. The EC, NEC, and SEs (torpedo) were collected at their stages and then quickly frozen in liquid nitrogen followed by the storage of -70 °C for subsequent RNA extraction.

### *EgHM*s expression analysis from available transcriptome data

We downloaded the transcriptome data of oil palm somatic embryogenesis stages (EC, NEC, and SE; Accession number: PRJNA699335) from the Sequence Read Archive (SRA) database of NCBI. The heatmap (http://www.omicshare.com/tools) was generated to calculate *EgHMs* expression levels in three different stages of somatic embryogenesis using RPKM values (RPKM= $\frac{{10}^{6}\mathrm{C}}{\mathrm{NL}\left/{10}^{3}\right.}$ [32].

### *EgHM*s gene expression analysis by quantitative real-time PCR

Total RNA was extracted from the different stages of calli (NEC, EC, and SE) during somatic embryogenesis using RNAprep pure Plant Kit (Tiangen, Beijing, China) by following the manufacturer’s instructions. The first-strand cDNA was synthesized using EasyScript® First-Strand cDNA Synthesis SuperMix (TransGen, Beijing, China) kit. The relative expression analysis of the 99 *EgHM* genes from the 109 identified *HM* genes was carried out by using the real-time qPCR method. The real-time qPCR was performed with SYBR® Select Master Mix (Thermo Fisher Scientific, Waltham, USA). The ABI QuantStudio™6 Flex quantitative real-time PCR instrument (Thermo Fisher, Waltham, USA) was used to analyze the data. The relative expression levels of 99 HM genes at different stages of somatic embryogenesis were calculated via 2^−ΔΔCt^ method. The qPCR primers for analyzing the expression of 99 HM genes were designed using QuantPrime qPCR primer designing tool (https:// quant prime. mpimp- golm. mpg. de/) and listed in Supplementary Table 3. All the qPCR reactions were performed with three biological and three technical replications. The oil palm actin gene (*EgActin1*) was used as an internal control to check the expression of *EgHM*s. The statistical significance at *p* < 0.05 was determined by using One-Way ANOVA.

## Conclusions

In conclusion, this is the first report on genome-wide analysis of histone modification genes (*HMT*, *HDM*, *HAT,* and *HDAC*) in oil palm. From this study, a total of 109 *EgHM*s were identified and analyzed candidate genes expression patterns during somatic embryogenesis of oil palm. Moreover, comprehensive information regarding their gene structure, motif, composition, chromosomal distribution, duplication events, evolutionary relationship with rice, and *Arabidopsis* was also reported. Furthermore, differential expression of various *EgHM*s at different stages of somatic embryogenesis was also elucidated through real-time PCR analysis indicating their potential involvement during the somatic embryogenesis of oil palm.

## Supplementary Information

**Additional file 1:** **Table 1.** List of EgHMs in oil palm genome.

**Additional file 2:** **Table 2.** Subcellular localaization prediction of 109 EgHM gene family.

**Additional file 3:** **Table 3.** List of qRT-PCR primer pairs used for realtive expression of EgHM gene family members in oil palm during somatic embryogenesis.

## Data Availability

The transcriptome data used in this study are available in the NCBI Sequence Read Archive (SRA) with BioProject accession number PRJNA699335. All data sets analyzed during this research are provided as Supplementary files and also included in the article. The data sets generated during this study are also available from the corresponding author on reasonable request.
